# Development of a Case-based Reading Curriculum and Its Effect on Resident Reading

**DOI:** 10.5811/westjem.2017.10.35117

**Published:** 2017-12-05

**Authors:** Anne M. Messman, Ian Walker

**Affiliations:** Sinai-Grace Hospital, Department of Emergency Medicine, Detroit, Michigan; Wayne State University, School of Medicine, Detroit, Michigan

## Abstract

Textbook reading plays a foundational role in a resident’s knowledge base. Many residency programs place residents on identical reading schedules, regardless of the clinical work or rotation the resident is doing. We sought to develop a reading curriculum that takes into account the clinical work a resident is doing so their reading curriculum corresponds with their clinical work. Preliminary data suggests an increased amount of resident reading and an increased interest in reading as a result of this change to their reading curriculum.

## BACKGROUND

Textbook reading plays a key role in the foundational knowledge base for many residents and is often incorporated into residency programs’ core curriculum. Many residency programs place residents on a reading schedule that is applied to all residents simultaneously without regard to what rotation the resident is currently on. This may force the resident to choose between completing their assigned reading versus reading about the patients they are caring for clinically.

## OBJECTIVES

Our objective in developing this curriculum was to increase interest in and compliance with reading. The goal is that with increased interest and compliance, learning will occur, as it has been shown that regular reading assignments increase in-training examination scores.^1^,^2^ Although residency programs may provide their residents with structured reading schedules, we could not identify any that correlate the reading schedule with what the resident is doing clinically. The learning theory guiding the creation of this reading curriculum is that of experiential learning: learning that is constructed from real-life experience.^3^ We felt that if the learner could connect their clinical work with the textbook reading, experiential learning would occur and the learner would be actively involved in the learning process. We sought to identify whether making this correlation resulted in residents reading more and whether they were more interested in reading.

## CURRICULAR DESIGN

We hold that there is a core body of knowledge, a core curriculum, that all interns should be expected to read. With this in mind, we reviewed the chapters offered in *Rosen’s Emergency Medicine – Concepts and Clinical Practice* (“Rosen’s”) textbook and divided them into two categories: chapters to be read during the intern year of residency and chapters that should be read during the second and third years of residency. This division of chapters was initially determined by the author (AM) in her role as associate program director and agreed upon by the other two members of program leadership. Once the list of “intern chapters” was created, we assigned chapters to specific rotations that the interns were on. For example, chapters pertaining to obstetrical or gynecological emergencies were assigned to the intern while she was on her obstetrics/gynecology rotation. Similarly, chapters that were pertinent to other off-service rotations were assigned during these respective rotations. All chapters left over were to be assigned to the resident while working clinically in the emergency department (ED). A similar process was undertaken for the second- and third-year chapters, with pertinent chapters assigned during off-service rotations and the rest assigned while working clinically in the ED. Residents were provided with a complete list of the chapters and their length, in pages, prior to the intervention so that they were aware that chapters had considerable variability in their length.

For those chapters designated to be assigned while working clinically in the ED, an Excel spreadsheet was created, accessible to the individual resident and all attending physicians. At the end of a shift, the attending physician would peruse this list with the resident and assign a chapter, pertinent to the clinical cases seen that day. All interns and residents played an integral role in the decision of what chapter was to be assigned, and generally attendings deferred to the residents’ decision of what chapter they would like to read. In the case that an attending forgot to assign a chapter, the resident could “self-assign” a chapter based on gaps in clinical knowledge that she perceived during that shift. Once the resident completed the reading assignment, he would go onto his spreadsheet and mark the chapter as “read” so that the same chapter would not be assigned more than once. Program leadership monitored the compliance and progress of the resident.

We used Google documents to store this information. Each resident had access to his own page; however, attending physicians had access to every resident’s page.

## IMPACT/EFFECTIVENESS

We surveyed residents prior to the implementation of the new curriculum and again three months after the initiation of the new curriculum. We obtained approval to disseminate this survey from our institutional review board. Residents were informed that the survey would be given two weeks before the surveys were administered so residents could keep track of their reading habits. The survey asked residents objective questions, such as how many hours per week they spent reading the Rosen’s textbook, as well as subjective questions, such as how beneficial they felt the reading was to their overall education. (See Appendix for a complete list of survey questions.)

We found statistically significant improvement via t-test in all parameters studied with the implementation of the new curriculum (Figure). These parameters included average number of hours per week spent reading the Rosen’s textbook (increased from 1.5 to 3.8, p = 0.002); how beneficial the Rosen’s reading was to their overall education (scale of 0–10 where 0 meant that reading was not beneficial at all and 10 that reading was extremely beneficial; increased from 5.4 to 6.7, p = 0.03); the impact their Rosen’s reading had on their clinical practice (scale of 0–10 where 0 meant that the reading made no impact on clinical practice and 10 means that reading has been extremely impactful on clinical practice, increased from 4.0 to 6.1, p = 0.007); and satisfaction with their current curriculum (scale of 0–10 where 0 meant the resident was completely unsatisfied with the curriculum and 10 means the resident was extremely satisfied with the curriculum, increased from 3.3 to 7.7, p = 0.0005).

## LIMITATIONS

Although all residents in this study used their Excel spreadsheet to track their reading, it would also be useful to measure to what degree the resident used the spreadsheet and how accurately it reflected the amount of reading actually completed, as residents may not have accurately recalled how much reading they actually did or may have provided false answers to please program leadership. Additionally, the study has inherent issues with generalizability as it was performed at a single institution that uses the *Rosen’s Emergency Medicine* textbook as its main reading source. Additionally, the residents were not sub-analyzed based on their post-graduate year and this may have provided useful data.

## CONCLUSION

Assigning textbook chapters that correlate with each resident’s clinical rotation is an educational innovation that could easily be adopted at any institution; it requires no funding or purchasing of any special software or textbooks. After our program adopted this innovation, we experienced an increased interest in reading and a renewed sense of responsibility among the residents regarding their education. Further studies would explore whether there is a subsequent increase in in-training examination scores or first-time board passage rates, and investigate *why* the residents experienced such significant increases in the parameters studied.

## Supplementary Information



## Figures and Tables

**Figure:**
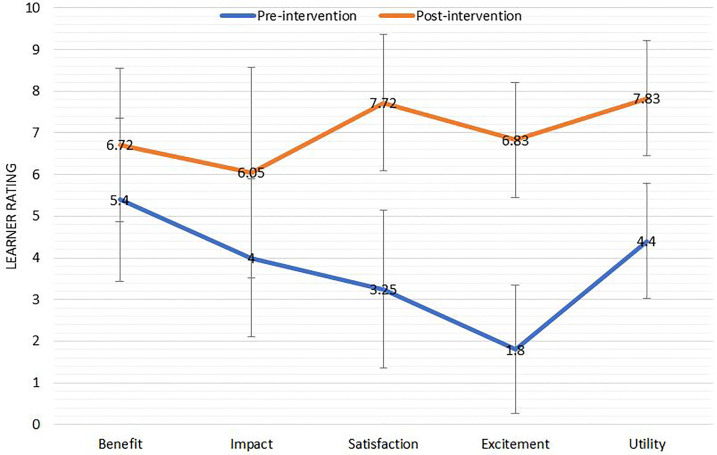
Learner ratings of the reading curriculum before and after the intervention. Average values are reported with error bars representing one standard deviation above and below the mean.
