# Supplementary material for: Development of a Case-based Reading Curriculum and Its Effect on Resident Reading
Source: West J Emerg Med. 2017 Dec 5;19(1):139–41. doi: 10.5811/westjem.2017.10.35117 (PMC5785182; doi:10.5811/westjem.2017.10.35117)
Supplement: Supplementary file 1 [file wjem-19-139-s001.docx]

**Appendix.** Survey questions

What is your current year of study in this residency program (i.e., what is your PGY level excluding years spent at other residency programs)?

How many hours per week, on average, did you spend reading from the Rosen’s textbook during the two-week period?

During the two-week study period, were you doing a clinical rotation in the emergency department? Please do not include ultrasound rotations, critical care rotations, etc.

How many hours per week did you spend, on average, on other educational resources (other textbooks, podcasts, blogs, etc,) during the two-week study period? Please do not include the time that you spent reading from the Rosen’s textbook.

On a scale of 0-10, how beneficial is reading from the Rosen’s textbook to your overall education, where 0 means that reading from the Rosen’s textbook is not beneficial at all and 10 means that reading from the Rosen’s textbook is extremely beneficial?

What percentage of the reading from the Rosen’s textbook do you think you have retained five days after reading? Please provide an answer from 0-100%.

On a scale of 0-10, how much impact has the **assigned** Rosen’s reading made on your clinical practice, where 0 means that your reading from the Rosen’s textbook has made no impact on your clinical practice and 10 means that your reading from the Rosen’s textbook has been extremely impactful on your clinical practice? (This refers to either your weekly Rosen’s reading assignment or your assignments based on the new curriculum, NOT reading from the Rosen’s textbook that you have pursued independently.)

On a scale of 0-10, how satisfied are you with the current Rosen’s textbook reading curriculum, where 0 means you are completely unsatisfied with the curriculum and 10 means you are extremely satisfied with the curriculum?

On a scale of 0-10, please rate how useful you feel the Rosen’s textbook reading curriculum is for your learning, where 0 means it is not useful at all for your learning and 10 means it is extremely useful for your learning.

On a scale of 0-10, please rate how excited you are to complete your **assigned** Rosen’s reading, where 0 means that you are not excited at all to complete your reading and 10 means that you are extremely excited to complete your reading.

Do you have any comments you would like to make?
